# Applying Nociplastic Pain Criteria in Chronic Musculoskeletal Conditions: A Vignette Study

**DOI:** 10.3390/jcm14041179

**Published:** 2025-02-11

**Authors:** Paraskevi Bilika, Jo Nijs, Evdokia Billis, Zacharias Dimitriadis, Achilleas Paliouras, Konstantina Savvoulidou, Nikolaos Strimpakos, Eleni Kapreli

**Affiliations:** 1 Clinical Exercise Physiology and Rehabilitation Research Laboratory, Department of Physiotherapy, School of Health Sciences, University of Thessaly, 35100 Lamia, Greece; apaliouras@uth.gr (A.P.); ksavvoulidou@uth.gr (K.S.); ekapreli@uth.gr (E.K.); 2 Pain in Motion Research Group (PAIN), Department of Physiotherapy, Human Physiology and Anatomy, Faculty of Physical Education & Physiotherapy, Vrije Universiteit Brussel, 1090 Brussels, Belgium; jo.nijs@vub.ac.be; 3 Chronic Pain Rehabilitation, Department of Physical Medicine and Physiotherapy, University Hospital Brussels, 1090 Brussels, Belgium; 4 Unit of Physiotherapy, Department of Health and Rehabilitation, Institute of Neuroscience and Physiology, Sahlgrenska Academy, University of Gothenburg, SE-405 30 Gothenburg, Sweden; 5 Physiotherapy Department, School of Health Rehabilitation Sciences, University of Patras, 26504 Patras, Greece; billis@upatras.gr; 6 Health Assessment and Quality of Life Research Laboratory, Department of Physiotherapy, School of Health Sciences, University of Thessaly, 35100 Lamia, Greece; zdimitriadis@uth.gr (Z.D.); nikstrimp@uth.gr (N.S.); 7 Division of Musculoskeletal & Dermatological Sciences, University of Manchester, Manchester M13 9PL, UK

**Keywords:** clinical criteria, nociplastic pain, pain phenotyping, reliability, validity, chronic musculoskeletal pain, sensitivity, specificity, pain assessment

## Abstract

**Background/Objectives:** The International Association for the Study of Pain (IASP) recently introduced clinical criteria and a grading system to identify nociplastic pain, marking a pivotal step toward improving diagnostic accuracy. This study aimed to evaluate the reliability and validity of the IASP criteria using clinical vignettes, assessing their effectiveness in identifying nociplastic pain in clinical settings. **Methods:** A reliability and diagnostic accuracy study was conducted using 32 clinical vignettes based on the literature and pre-existing clinical data. The vignettes represented patients with and without the characteristics of nociplastic pain and were reviewed independently by two expert physiotherapists. Inter-rater and intra-rater reliability were evaluated with a 1-month interval between assessments. Criterion validity was analyzed by comparing the IASP criteria against the standardized vignettes as the reference standard. Sensitivity, specificity, and predictive values were calculated to assess diagnostic accuracy. **Results:** The IASP criteria demonstrated moderate-to-perfect intra-rater agreement (κ = 0.71–1.00, *p* < 0.05) and weak-to-perfect inter-rater agreement (κ = 0.52–1.00, *p* < 0.05). Criterion validity was moderate (κ = 0.68), with strong specificity (89.0%) and moderate sensitivity (69.0%). Positive and negative predictive values were high at 81.8% and 81.0%, respectively, supporting the criteria’s accuracy in identifying and excluding nociplastic pain. **Conclusions:** The IASP criteria for nociplastic pain exhibited satisfactory reliability and criterion validity in this preliminary study, particularly after initial rater familiarization. Future research should evaluate their application in real-world clinical settings, explore concurrent and prognostic validity, and involve a broader range of raters to enhance generalizability.

## 1. Introduction

In 2016, the International Association for the Study of Pain (IASP) introduced nociplastic pain, as the third pain phenotype, in addition to nociceptive and neuropathic pain mechanisms [1,2].

Nociplastic pain is defined as “pain that arises from altered nociception despite no clear evidence of actual or threatened tissue damage causing the activation of peripheral nociceptors or evidence for disease or lesion of the somatosensory system causing the pain”. Central Sensitization (CS) is considered the major underling mechanism of nociplastic pain [3,4]. CS concerns the modified central nervous system (CNS) sensory processing, dysfunction of the mechanisms of pain inhibition, increased activity of the pain facilitatory pathways, and long-term potentiation of the neural synapses in the anterior cingulate cortex [5]. When CS is present, hypersensitivity to pain is observed due to enhanced signaling within the CNS, and pain is no longer protective but arises spontaneously [3]. Many studies have identified the presence of CS in patients with a variety of musculoskeletal disorders [2,6,7] such as chronic low back pain [8], chronic shoulder pain [9], knee osteoarthritis [10,11,12], whiplash [13], and fibromyalgia [2]. It should be noted, however, that the prevalence of central sensitization varies significantly, ranging from very low in cases of shoulder pain to a high level in cases such as fibromyalgia [14], thus making further research into this population more challenging.

Nociplastic pain may provide an explanation for unexplained chronic musculoskeletal pain (CMP) that is beyond tissue damage or pathology [15,16], unlike nociceptive pain, which is often associated with observable tissue damage or inflammation, and neuropathic pain, which can typically be traced to. In order to manage pain, it seems crucial to recognize patients with nociplastic pain, since the literature suggests that mechanism-based pain treatment is better than treatments focusing solely on symptoms or specific diseases [17,18,19]. The recognition of nociplastic pain poses a significant challenge for clinicians due to its subjective nature and the lack of clear peripheral pathology [4]. As a result, many patients often encounter skepticism or doubt from healthcare providers [20]. This diagnostic ambiguity can lead to underdiagnosis or misdiagnosis, emphasizing the importance of structured diagnostic criteria. Moreover, recognizing and accurately diagnosing nociplastic pain can help clinicians avoid ineffective treatments. Traditional analgesics such as non-steroidal anti-inflammatory drugs (NSAIDs) and opioids are generally less effective in managing nociplastic pain and may even exacerbate symptoms in some cases [21,22]. Understanding the central nature of nociplastic pain can prevent the overuse of these medications, reducing the risk of side effects and dependency. The evaluation of pain mechanisms is increasingly recognized as a vital component of understanding the pain experience, though its clinical application remains underexplored. Studies demonstrate that while pain mechanism evaluation holds potential for tailored interventions, it is not frequently utilized in practice [23].

For instance, Arendt-Nielsen and Yarnitsky [24] discuss how quantitative sensory testing offers valuable insights into pain mechanisms in clinical settings, but note its limited application due to practical barriers. Similarly, Rowbotham [25] highlights the necessity of the pre-trial testing of pain mechanisms in clinical trials, underscoring its potential but also its rarity in regular clinical use. Physical therapists also acknowledge the utility of pain mechanism assessments but often struggle with integrating them into standard orthopedic practices [23].

The clinical criteria for nociplastic pain, as developed by Kosek et al. and endorsed by the IASP in 2022, provide a critical framework to address this issue [26,27]. These criteria focus on the presence of central sensitization features—such as widespread pain, hypersensitivity to sensory stimuli, and associated symptoms like fatigue and cognitive difficulties—while ruling out nociceptive or neuropathic origins. So far, there has been a detailed analysis of the criteria and their application clinically [26,27].

Guidelines such as the Cancer Pain Phenotyping (CANPPHE) Network recommendations for cancer survivors [17,28] and the Back Pain Phenotyping Consortium (BACPAP) guidelines for chronic low back pain [8] illustrate how these criteria are applied in specific clinical populations. These recommendations underscore the importance of integrating multidimensional tools, including body maps, symptom questionnaires, and sensory assessments, into routine practice. By adopting these tools, clinicians can more accurately phenotype nociplastic pain, ensuring early identification, avoiding unnecessary testing, and tailoring treatment plans to address CS effectively. However, the reliability and validity of the IASP criteria and the grading system for nociplastic pain have not been tested. The assessment of the reliability and validity of the IASP criteria and grading system for nociplastic pain is vital, as it confirms their consistency across different assessors, validates their ability to accurately identify nociplastic pain, and ensures their practical utility in real-world settings. This enhances clinicians’ confidence in using the criteria, improves diagnostic accuracy, and supports personalized treatment strategies.

As reported by Nijs et al., clinical vignettes are valuable for assessing the reliability and validity of criteria for identifying nociplastic pain. Vignettes have been used in many social studies [29] but also in medical studies for diagnosis, clinical reasoning, and disease management [30,31]. They are developed in an appropriate way to give the necessary information to the readers to form their own point of view based on the knowledge and experience they have [32]. Scenario design for vignettes is not arbitrary; it is carefully crafted to simulate real cases and capture the attention of readers. Moreover, they adhere to specific rules to ensure internal validity, including the use of pilot studies, the use of evidence-based data, the inclusion of real patient data, and their development by experts [33]. Unlike studies involving real patients, which are influenced by variability in patient presentation, vignettes enable researchers to control confounding variables and ensure consistency across evaluations. Clinical vignettes allow for identical presentation of scenarios to multiple assessors, eliminating the variability associated with live patient interactions. Furthermore, vignettes can be designed to reflect situations or combinations of factors that are challenging to study in real-world conditions due to their low prevalence.

The purpose of the study was to examine the reliability, validity, diagnostic accuracy, and inter-rater consistency of the IASP clinical criteria for nociplastic pain using vignettes. The primary advantage of utilizing vignettes as a sample in this reliability study is that they could enable the inclusion of an adequate sample size featuring patients with a diverse range of conditions. This study is a preliminary step toward a more comprehensive evaluation of the IASP clinical criteria.

## 2. Materials and Methods

**Figure 1:**
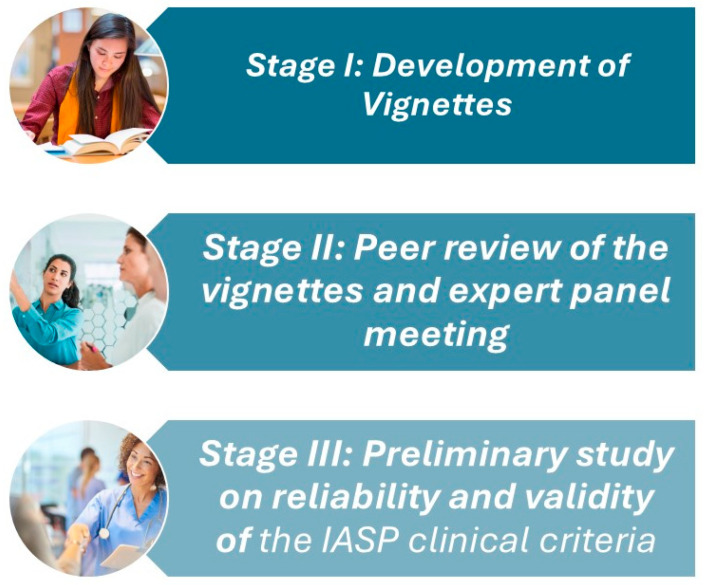
Study procedure.

This study was conducted in three stages (Figure 1):

Stage I: Development of clinical vignettes based on the existing literature and pre-existing clinical data to represent patients with and without the characteristics of nociplastic pain.

Stage II: Peer review of the vignettes by two experts in the field to ensure realism and completeness of content.

Stage III: A preliminary study assessing the inter-rater and intra-rater reliability, as well as the criterion validity, of the IASP clinical criteria and grading system using the validated vignettes.

### 2.1. Stage I: Development of Vignettes

A researcher (PB) with postgraduate studies, 5 years of research on pain, and clinical experience managing patients CMP developed the vignettes. The researcher conducted a comprehensive search in the PubMed database for the period of 2014–2018, to identify the factors (clinical characteristics, signs etc.) associated with nociplastic pain and CS. The characteristics of the vignettes about pathology, distribution of pain, total questionnaire scores, and demographics (age, occupation, and educational level) were data from our previous study [34]. The names and hobbies were chosen randomly. The vignettes were based on 5 different factors: (1) pain disproportionate to the nature and extent of injury or pathology [6,35,36,37,38], (2) diffuse/non-anatomic areas of pain [35,37,39], (3) Central Sensitivity Questionnaire Score over 40 or presence of hypersensitivity to bright light, sound, smell [39,40], (4) hyperalgesia/tenderness on palpation [35,36,37], and (5) gender [41,42,43].

The combination of factors determined the clinical characteristics of patients in vignettes (factor design) [44,45]. The vignettes were based on real patients with CMP in 4 possible anatomic areas: (A) neck, (B) shoulder, (C) low back and (D) knee. The vignettes were classified into these 4 categories (8 vignettes for each anatomic area). The possible combinations of factors classified in each anatomic area were random with the help of the web application https://www.random.org/ (accessed on 18 September 2024). All vignettes contained the necessary information for the classification and presented a uniform structure, enabling readers’ understanding. The assessment section presented information based on accepted clinical practice and included subjective and objective examination as in the study by Dewitte et al. [46]. An example vignette is attached to Supplementary Materials S1.

### 2.2. Stage II: Peer Review of the Vignettes and Expert Panel Meeting

In Round 1 of peer review, the vignettes were evaluated independently by 2 expert physiotherapists (EK, EB) with clinical experience in patients with CMP (17 and 18 years of experience, respectively), academics that teach pain neurophysiology in higher education, both undergraduate and postgraduate levels. During the evaluation of the vignettes, they completed a specially designed questionnaire in order to assess the realism, simplicity, understanding, and completeness of the vignettes [47]. In the first part of the questionnaire, the experts scored on a Likert scale of 5 points. In the second part, the raters answered the final question “Does this vignette include a patient with nociplastic pain?” with a “no”, “yes” or “unsure” (Supplementary Material S2). The experts had the opportunity to suggest changes to the wording of the vignettes that they believed might aid discrimination between nociplastic pain or non-nociplastic pain. The involvement of the experts contributed to the improvement of the content of the vignettes [48].

In Round 2, a meeting was held between all three physiotherapists (experts and researcher) regarding the evaluation of vignettes. Vignettes that were not considered appropriate or were difficult to use in discriminating between the categories “nociplastic pain” and “non-nociplastic pain” were analyzed extensively for modification. Wording changes were accepted if approved by a majority of panelists (2 out of 3). At the end of the meeting, the final vignettes were classified into the “nociplastic pain” category or into the “non-nociplastic pain” category with the agreement of experts (consensus diagnosis).

### 2.3. Stage III: Preliminary Study on Reliability and Validity of the IASP Clinical Criteria

#### 2.3.1. Raters’ Selection

The target population was musculoskeletal physiotherapists. An open call was issued. The invitation was posted on the website of the University and Social Pages. The inclusion and exclusion criteria are presented in Table 1.

The inclusion criteria for raters in this study were designed to ensure a high level of knowledge and clinical reasoning skills in central sensitization and nociplastic pain. With the selection of highly experienced raters, we aimed to offset the variability in reliability.

#### 2.3.2. Procedure

Physiotherapists who responded to the call received a survey package. It included an introductory letter outlining the aims of the study, a questionnaire and a consent form that allowed the physiotherapist to give reasons for non-participation (inability to complete the questionnaire or simple refusal to participate). After an informed written consent, which allowed for revocation at any time without the need for explanation, the selection of physiotherapists according to the inclusion and exclusion criteria was conducted. Out of the seven physical therapists who expressed interest, only two met the participation criteria. The two selected physiotherapists (raters) participated in a 3 h familiarization with the clinical criteria of IASP. The familiarization took place on the same day in the presence of all selected physiotherapists (raters) and included the detailed explanation of the criteria and the evaluation of some vignettes as examples to familiarize themselves with the procedure. During the familiarization phase, informal assessments, including case discussions and mock evaluations of example vignettes, were conducted to gauge the raters’ understanding of the IASP criteria. Both raters demonstrated proficiency in applying the criteria by achieving a consensus with the study lead in these trial assessments. This process ensured a consistent baseline of knowledge prior to the study. One day later, the raters received the 32 vignettes in random order and were asked to read them and complete a set of questions (Supplementary Material S3), which included the IASP clinical criteria. All questionnaires were given in electronic form to facilitate data collection. The same procedure was repeated after one month. The vignettes were given in the same order as they were given in the previous assessment. The re-evaluation (test-retest) was conducted after a period of one month. While conventional studies often perform re-evaluations after a shorter interval, typically around one week, the utilization of vignettes in our study afforded us the opportunity to extend this timeframe to one month. This decision was made to mitigate the potential impact of memory recall on our results, ensuring the reliability of the test-retest assessments.

The questionnaires were administered using Microsoft Forms (MS Forms). This platform was selected for its user-friendly interface MS Forms which offers the advantage of real-time data tracking and automatic data storage in Microsoft Excel, ensuring the efficient management of survey responses. Furthermore, its compatibility with various devices enables respondents to complete the questionnaires conveniently from any location, enhancing accessibility and participation rates.

### 2.4. Statistical Analysis

The calculation of the required number of vignettes was made based on the statistical index κ that was used to investigate reliability and the fact that the answers were given in a dichotomized form (nociplastic pain or non-nociplastic pain—table 2 × 2). Based on the hypothesis κ_1_ = 0.5 and κ_2_ = 0.9, to achieve a statistical power of 80% and a level of α = 0.05 and taking into account a probability of data loss of 10%, the proposed number of vignettes was 30. Having 5 factors with 2 levels each (level = possible choices: male or female for gender, yes or no for the other factors), the possible combinations were 25, i.e., 32 different combinations or 32 clinical vignettes.

The agreement was a measure of reliability when assessing the categorical responses [51]. Inter-rater reliability was examined by evaluating the agreement between raters. Test–retest reliability was examined by assessing the initial evaluation of the raters with their evaluation after one month. Criterion validity assesses the extent to which the assessment results obtained from a measurement instrument correlate with an external criterion [52]. Criterion validity was assessed by estimating the agreement between the results of the IASP clinical criteria (which the raters used) with the priori classification of the vignettes.

To assess criterion validity, raters’ responses were converted from three possible responses (no, possible, probable nociplastic pain) to two (nociplastic pain or non-nociplastic pain). Vignettes assigned to “probable” or “possible nociplastic pain” were scored as “nociplastic pain”. Given that the IASP criteria and the priori classification of the vignettes provide categorical (nominal) data, intra-rater reliability, inter-rater reliability, and criterion validity were examined using Cohen’s kappa [53,54] with corresponding confidence intervals and the agreement rate (Supplementary Materials S4). This approach was chosen to align with clinical management practices, where similar treatment strategies are employed for probable and possible nociplastic pain.

Moreover, the sensitivity, specificity, positive predictive value (PPV), and negative predictive value (NPV) were calculated. Specificity indicates the proportion of truly negative cases that the test correctly identifies as negative. Sensitivity represents the proportion of truly positive cases that the test correctly identifies as positive. It measures how well the test detects the actual positives. PPV is the likelihood that individuals with a positive test result truly have the condition. NPV is the likelihood that individuals with a negative test result truly do not have the condition [55]. All data analyses were performed with IBM SPSS Statistics for Windows, Version 26.0. The significance level was set to α = 0.05.

## 3. Results

According to the experts’ assessments, all of the vignettes were rated as easy to read and clear, receiving a score of 5 out of 5 on the Likert Scale. Furthermore, a significant percentage of the vignettes (85.3%) was considered to be extremely realistic (59.4%) or realistic enough (25.9%), whereas the 93.7% of the vignettes were considered extremely thorough (54.7%) or sufficiently thorough (39%). These findings suggest that the vignettes were well-crafted and deemed to be both accurate and informative representations of clinical cases. After undergoing evaluations by the experts, four of the vignettes were rated as having partial completeness and three were moderately realistic. These vignettes were subsequently redesigned based on the recommendations provided by the experts. This process aimed to improve their completeness and realism to ensure that they accurately represented clinical cases and provided sufficient information for clinical reasoning.

The experts evaluated the 32 vignettes and concluded that 12 out of them involved patients with nociplastic pain. The agreement between experts was moderate (κ = 0.64). The experts disagreed with the verdict in 6 vignettes. Eight vignettes required revision, such as changes in pain distribution, medication, level of functioning, and physical examination. More information is provided in Table 2.

The final version of the vignettes included 32 CMP patients with pain in the neck, shoulder, low back, or knee. In Table 3, the characteristics of vignettes were presented. A statistical comparison was conducted between the nociplastic pain and non-nociplastic pain groups (Table 3). Significant differences were observed in CSI scores (*p* = 0.03) and pain intensity (*p* = 0.04), with the nociplastic pain group showing higher scores in both parameters. Gender distribution also differed significantly, with fewer men in the nociplastic pain group (*p* = 0.02). Further analysis indicated that specific vignette characteristics may influence diagnostic accuracy. Vignettes featuring diffuse pain distribution (more than eight areas on the body chart), pain intensity greater than 5, or a CSI total score of ≥40 were more consistently classified as nociplastic pain. Specifically, 8 out of 12 cases (66.7%) classified as nociplastic pain exhibited pain in more than eight body regions, compared to 7 out of 20 cases (35%) classified as non-nociplastic pain. Additionally, 10 out of 12 cases (83.3%) with a pain intensity >5 were categorized as nociplastic pain, whereas 13 out of 20 cases (65%) were classified as non-nociplastic pain. In contrast, age did not appear to significantly impact diagnostic accuracy.

### 3.1. Raters and Training

Two physiotherapists (mean age: 28 years) were selected to rate the vignettes. The raters met the inclusion criteria and achieved the highest score (19/19) on the NPQ questionnaire. The raters completed their post-graduate studies and received special training in pain mechanism.

### 3.2. Intra-Rater Reliability

Intra-rater reliability was examined separately for each criterion and for the overall decision (non-nociplastic pain, possible nociplastic pain, probable nociplastic pain). The agreement between the results of the IASP clinical criteria for each rater was strong to perfect (κ = 0.81–1.00), except one question which showed moderate agreement (κ = 0.71) (Table 4).

### 3.3. Inter-Rater Reliability

The two raters separately assessed the vignettes twice. The agreement between the raters was estimated as strong to almost perfect (κ = 0.81–1.00) in the first assessment after the training, and as weak to almost perfect (κ = 0.52–1.00) in the second assessment (Table 5).

### 3.4. Criterion Validity

The agreement between the results of the IASP clinical criteria (which the raters used) and the a priori classification of the vignettes was moderate (κ = 0.60) for both of raters in the first assessment and weak (κ_1_ = 0.42, κ_2_ = 0.54) in the second assessment.

### 3.5. Specificity and Sensitivity

Clinical criteria showed high specificity (89.0%), moderate sensitivity (69%), high positive predictive value (PPV = 81.8%), and high negative predictive value (NPV = 81%), indicating that the criteria are highly reliable for correctly identifying both positive and negative cases of nociplastic pain.

## 4. Discussion

The current study is a first attempt to examine an easy-to-use grading system based on the IASP criteria to guide healthcare professionals’ clinical decisions regarding the identification of patients with nociplastic pain. Although the criteria and the updated grading system for nociplastic pain have been established since 2021 [26], there are no published studies investigating the reliability or validity of these criteria. According to the current study’s findings, the IASP criteria demonstrated satisfactory reliability and criterion validity, particularly in the first assessment after raters’ familiarization.

The intrarater reliability was strong to perfect in all criteria but one that yielded moderate agreement. The agreement between raters was estimated as moderate to almost perfect in the first assessment, and as weak to almost perfect in the second assessment. As mentioned, an important limitation of the criteria is that the clinician should decide whether the pain mechanism is entirely nociceptive or neuropathic [26]. In the present study, however, moderate-to-perfect agreement was found when the vignettes were rated by the same rater or different raters immediately after training. On the other hand, after a month, the inter-rater agreement on the predominance of nociceptive pain was weak.

The decrease in inter-rater agreement during the second evaluation could be attributed to several factors. One primary reason is the time elapsed between training and the second assessment. Given that the physiotherapists were not previously familiar with the IASP criteria, their retention and application of the criteria may have been affected over time, leading to inconsistencies in classification. The initial assessment was conducted shortly after training, when the raters’ understanding of the criteria was likely more precise and aligned, contributing to higher agreement. However, after one month, variations in recall, interpretation, or subtle shifts in diagnostic reasoning could have contributed to the discrepancies in their evaluations.

Additionally, the process of re-evaluating the same vignettes may have introduced cognitive fatigue or reduced attentiveness, particularly if raters subconsciously recalled previous responses rather than fully reassessing each case. This phenomenon, known as rater drift, occurs when evaluators deviate from their initial application of criteria over time, leading to decreased consistency. Furthermore, the subjectivity inherent in distinguishing between nociceptive and neuropathic pain mechanisms [26] may have exacerbated variability in classification, as different raters may weigh specific diagnostic elements differently in subsequent assessments.

Rater drift, or the gradual deviation in scoring patterns over time, is a well-documented phenomenon that can affect inter-rater reliability in various assessment contexts. Studies have shown that time-based rater variance can introduce inconsistencies, particularly when raters are not consistently recalibrated or when the evaluation process requires complex decision-making [56]. One of the primary reasons for rater drift is the time elapsed between training and subsequent assessments, leading to a decline in consistency due to memory decay or evolving subjective interpretations of the criteria. Şevgin and Şata [57] highlight that changes in rater perception over time can significantly impact the reliability and validity of performance assessments, reinforcing the importance of refresher training to maintain consistency.

Another contributing factor is cognitive fatigue, which can emerge when raters are required to reassess the same cases after an extended period. Aslett [58] discusses how examiner reliability can fluctuate due to external influences, including subjective variables and reduced attentiveness during later evaluations.

Another point is the inter-rater agreement of the criterion regarding the presence of nociceptive pain. According to the IASP terminology, nociceptive pain is defined as “pain that arises from by actual or threatened damage to non-neural tissue and is due to the activation of nociceptors” [4]. Therefore, patients who presented the pain experience and level of functioning proportionate with the underlying pathology or injury were classified as patients with nociceptive pain [27,39]. Also, the presence of pain with aggravating and relieving factors was considered an element of the presence of nociceptive pain [35].

Due to the lack of gold standard for identifying nociplastic pain, we set up an expert panel and used the vignette method. Expert panels utilized a combination of evidence and clinical expertise, with transparent opinions that were subjected to critical appraisal. This addressed the primary concern with relying solely on expert opinion and had the added benefit of incorporating information that is more closely aligned with clinical practice and creating a stronger connection with research evidence [59]. Therefore, in the present study the standardized vignettes were considered a reference standard. The development and use of vignettes is a very useful method for both training clinical therapists in a variety of situations and as a tool for testing the reliability and validity of algorithms such as the IASP clinical criteria and grading system for nociplastic pain. The development of clinical vignettes based on the literature by a researcher with previous experience and knowledge about CMP and CS, the incorporation of real data from previous study, and the revision of vignettes by experts in the field of CMP were all important aspects to strengthen the content validity of the vignettes.

While there is no definitive gold standard for diagnosing nociplastic pain, our findings on specificity (89%) and sensitivity (69%) align with prior studies that validated similar pain phenotyping tools. The sensitivity and specificity of the CSI as a diagnostic tool for central sensitization syndromes have been examined in multiple studies. Neblett et al. (2013) established clinically significant values for the CSI and demonstrated its utility in identifying central sensitivity syndromes [40]. Later studies further confirmed that the CSI effectively distinguishes between patients with and without central sensitization, reinforcing its diagnostic potential [60]. Previous studies found that in musculoskeletal pain populations, the CSI demonstrated moderate-to-high sensitivity (31–92.3%) and specificity (75–93.3%) when compared to healthy individuals, suggesting that while the CSI is useful for identifying central sensitization, its sensitivity may vary depending on the patient population [61,62,63,64]. Discrepancies in sensitivity across studies may arise due to variations in study design, differing definitions of nociplastic pain, and differences in patient populations.

The new IASP clinical criteria and grading system for nociplastic pain can appropriately guide clinicians in decision-making, promoting precision pain medicine, whereby patients are classified into categories based on their clinical characteristics, prognosis, and other factors in order to formulate the appropriate personalized treatment for them. The present study is of high clinical importance as it reinforces the application of the new IASP clinical criteria and grading system for nociplastic pain in clinical practice, demonstrating the satisfactory repeatability and criterion validity of the identification system for patients with nociplastic pain. The grading system exhibits favorable specificity and positive predictive value, indicating its reliability in correctly identifying individuals without the condition. However, its sensitivity, while moderate, suggests potential for missed positive cases. Furthermore, the findings of this study suggest that while the IASP criteria for nociplastic pain demonstrate strong intra-rater reliability, inter-rater agreement may vary. Given the variability observed in inter-rater reliability, incorporating structured clinical checklists or decision-support tools based on the IASP criteria can enhance consistency in assessments. Regular training sessions and case discussions among healthcare professionals can help align the interpretations and application of the criteria, minimizing diagnostic discrepancies. To mitigate rater drift in future studies, implementing periodic recalibration sessions, shorter reassessment intervals, and structured feedback mechanisms can help maintain inter-rater reliability and prevent divergence in rating tendencies over time. Clinicians should consider these performance characteristics when interpreting test results and making clinical decisions.

### 4.1. Limitations

This study acknowledges some limitations. First, the evaluation of sensitivity, specificity, PPV, and NPV is constrained by the absence of a universally accepted reference standard for diagnosing nociplastic pain. Currently, the IASP criteria serve as the most established tool for identifying this patient cohort, offering clinicians a common reference point.

Vignettes provide insight into standardized research assessments but lack clinical evaluation complexity. However, vignettes are advantageous in reliability studies despite this limitation, since they facilitate the inclusion of a diverse patient population, such as patients with a wide range of conditions and symptoms or rare ones. Furthermore, it reduces the risk of sample loss during re-assessment, a critical factor in ensuring success in reliability studies.

In the context of inter-rater agreement, it is often preferable to employ more than two raters to obtain a more robust estimate of agreement. With only two raters, distinguishing between chance and true agreement poses a challenge in the current study. Nevertheless, the use of an adequate number of vignettes based on power calculation (in our case 32) partially alleviates this limitation by providing a broader scope for evaluation.

The current study included raters with expertise and familiarity with nociplastic pain. Several factors influenced this decision. The concept of nociplastic pain is not yet well understood by clinicians who manage patients with CMP. The selection of these specific raters allowed us to assess the reliability and validity of the vignettes in a context in which the raters had a thorough understanding of the neurophysiological mechanisms underlying nociplastic pain, in a somewhat ideal situation. This allowed us to identify potential issues and refine our methodology before expanding the study. While this focused approach enhanced reliability, we acknowledge that a larger group of raters would provide a more comprehensive evaluation of inter-rater variability. A one-month interval for test–retest analysis was chosen to minimize recall bias while providing sufficient time for potential changes in interpretation. Although clear instructions and detailed explanations for each criterion were provided, as described in the assessors’ evaluation forms, the variability in reliability may be linked to the time elapsed since training (e.g., memory loss or changes in criteria interpretation). This issue was beyond the scope of the study. Future studies could investigate in greater detail the factors influencing the tool’s reliability.

Finally, while the conversion of the three response options into two categories for statistical analysis facilitated the differentiation between nociplastic and non-nociplastic pain, it may have introduced a potential imbalance in the results. Specifically, collapsing the “probable” and “possible nociplastic pain” categories into a single group could skew the findings toward a bias for nociplastic pain. In spite of the choice to align with clinical management practices, similar treatment strategies are often employed for probable and possible nociplastic pain. To address this limitation, future studies should explore alternative analytical methods.

### 4.2. Future Research

One potential refinement to improve the diagnostic accuracy of the IASP criteria involves incorporating an assessment protocol for allodynia in a remote painful area [27]. Allodynia, defined as pain in response to a normally non-painful stimulus, is often associated with central sensitization. Future validation studies should explore the diagnostic value of remote allodynia testing in distinguishing nociplastic pain from other pain mechanisms. By systematically incorporating this evaluation into the clinical framework, clinicians may be able to enhance the sensitivity of the criteria while maintaining their specificity.

Future research should involve a broader range of healthcare professionals, including those from different clinical specialties (physiotherapists, rheumatologists, and pain specialists) and varying levels of experience (from trainees to senior clinicians). Conducting similar studies with an expanded group of raters would strengthen the external validity of the IASP criteria and facilitate their integration into routine clinical practice. Furthermore, future research could focus on real patients instead of vignettes. Other chronic pain patients, such as cancer survivors, could be assessed using the present methodology [18].

Vignettes could be used as a pedagogical tool to train clinicians in identifying nociplastic pain. These vignettes can be integrated into continuing education workshops or included in certification programs focused on pain management. For example, clinicians could complete vignette-based assessments before and after training to evaluate their diagnostic improvement. Additionally, online platforms could host interactive vignette libraries, allowing clinicians to practice applying the IASP criteria in diverse clinical scenarios. These tools would enhance the accessibility and effectiveness of nociplastic pain education.

## 5. Conclusions

This study represents a preliminary step toward a more comprehensive evaluation of the applicability of IASP clinical criteria that can assist healthcare professionals in identifying patients with nociplastic pain. Strong to almost perfect agreement in repeated measurements in intrarater and between raters was found, although some criteria showed weaker agreement during the re-assessment after one month period. Overall, the IASP clinical criteria and grading system for nociplastic pain provide a useful tool for pain phenotyping, allowing for personalized treatment plans. The present study contributes to the growing body of evidence supporting the use of the IASP criteria and grading system in clinical practice. To further establish the clinical utility of the IASP criteria, future research should focus on testing their validity in real-world clinical settings. Additionally, integrating these criteria into standardized diagnostic algorithms could enhance their applicability in routine practice. Further studies involving a broader range of healthcare professionals and diverse clinical contexts will be essential to refine and optimize the implementation of nociplastic pain assessment in daily clinical care.

## Figures and Tables

**Table 1:** Inclusion and exclusion criteria.

Inclusion	Exclusion
Previous participation in courses or seminars on CMP	Those who cannot understand, read, and write in Greek
At least 2 years of clinical experience in the management of CMP	Those who have not been qualified as physiotherapist yet
Strong understanding of pain neurophysiology, achieving a score of 17/19 on the NPQ, allowing for up to 10% deviation [49,50]	Those who did not have specialized knowledge in CMP and CS
Postgraduate certification in physiotherapy	
Contribution to studies (clinical trials/systematic reviews etc.) related to pain	
Unfamiliarity with the IASP clinical criteria for identifying patients with nociplastic pain	

NPQ = Neurophysiology of Pain Questionnaire, CMP = chronic musculoskeletal pain, CS = central sensitization, IASP = International Association of the Study of Pain.

**Table 2:** Experts’ decisions.

Vignette	Expert 1	Expert 2	Final Decision	Changes
1	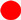	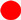	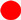	-
2	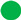	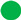	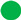	-
3	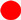	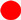	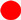	-
4	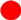	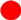	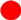	-
5	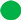	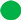	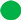	-
6	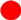	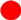	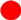	-
7	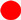	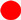	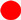	-
8	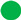	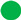	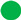	Change in medication and pain distribution
9	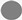	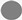	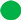	Change in CSI score
10	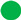	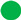	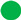	-
11	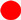	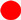	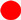	-
12	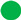	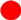	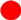	Changes in physical examination
13	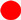	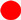	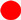	-
14	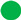	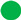	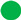	-
15	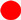	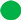	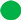	No sensitivity in palpation
16	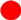	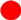	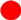	-
17	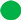	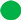	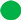	-
18	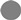	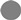	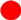	Change in medication and physical examination
19	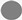	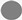	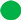	Add sensitivity in palpation
20	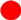	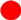	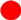	-
21	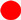	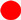	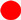	-
22	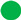	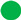	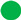	-
23	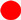	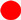	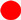	-
24	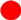	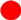	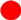	-
25	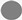	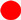	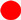	Expert 1 changed his verdict
26	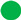	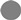	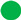	Change in pain distribution
27	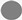	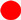	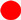	Expert 1 changed his verdict
28	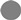	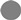	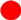	No sensitivity in palpation
29	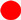	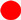	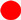	-
30	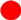	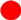	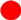	-
31	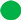	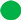	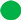	-
32	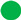	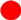	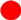	Expert 1 changed his verdict

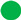
 nociplastic pain 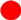 non-nociplastic pain 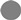 I’m not sure.

**Table 3:** Characteristics of the vignettes.

Sample Characteristic	Non-Nociplastic Pain Phenotype *n* = 20	Nociplastic Pain Phenotype *n* = 12	*p*-Value
Age (SD)	47.8 (11.47)	45.92 (12.03)	0.83
Pain Intensity (SD)	6.50 (1.28)	7.08 (1.24)	0.04
CSI ^1^ (SD)	34.75 (10.99)	37.33 (12.19)	0.03
Men N (%)	13 (65.0)	3 (25.0)	0.02
Neck Pain (%)	6 (30.0)	2 (16.7)	-
Shoulder Pain (%)	4 (20.0)	4 (33.3)	-
Low Back Pain (%)	5 (25.0)	3 (25.0)	-
Knee Pain (%)	5 (25.0)	3 (25.0)	-

^1^ CSI = Central Sensitization Inventory.

**Table 4:** Intra-rater agreement of the criteria.

Criteria	Rater 1	Rater 2
Regional (rather than discrete) distribution	0.81	1.00
Is there evidence that nociceptive pain is present?	0.89	0.82
Is nociceptive pain entirely responsible for the pain?	0.94	0.81
Is there evidence that neuropathic pain is present?	0.71	0.93
Is neuropathic pain entirely responsible for the pain?	0.87	1.00
Evoked pain hypersensitivity phenomena	0.88	0.94
There is a history of pain hypersensitivity	0.86	0.86
Presence of comorbidities	0.94	0.94
Final Decision	0.95	0.85

The colors indicate the strength of the agreement based on Supplementary Materials S4.

**Table 5:** Inter-rater agreement of the criteria in the first and the second assessment.

Criteria	First Assessment	Second Assessment
Regional (rather than discrete) in distribution	0.81	0.52
Is there evidence that nociceptive pain is present?	0.80	0.75
Is nociceptive pain entirely responsible for the pain?	0.88	0.58
Is there evidence that neuropathic pain is present?	0.92	0.72
Is neuropathic pain entirely responsible for the pain?	0.84	0.81
Evoked pain hypersensitivity phenomena	1.00	0.93
History of pain hypersensitivity	0.93	1.00
Presence of comorbidities	1.00	1.00
Final Decision	0.95	0.70

The colors indicate the strength of the agreement based on Supplementary Materials S4.

## Data Availability

The data provided in this research can be obtained upon request from the corresponding author.

## Supplementary Materials

The following supporting information can be downloaded at: https://www.mdpi.com/article/10.3390/jcm14041179/s1. S1: Example of a vignette; S2: Vignettes Assessment Form (Experts); S3: Vignettes Assessment Form (Raters); S4: Interpretation of Cohen’s kappa for calculating agreement (adapted from McHugh [48]).
