# Supplementary material for: Applying Nociplastic Pain Criteria in Chronic Musculoskeletal Conditions: A Vignette Study
Source: J Clin Med. 2025 Feb 11;14(4):1179. doi: 10.3390/jcm14041179 (PMC11856051; doi:10.3390/jcm14041179)
Supplement: Supplementary file 1 [file jcm-14-01179-s001.zip › jcm-3434491-supplementary.pdf]

S1: Example of a vignette

Vignette No. 20

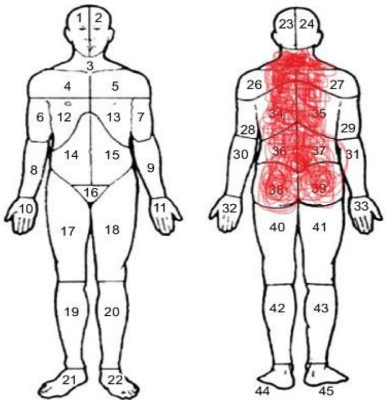

Patient Information

Mr. Raphael is a 50-year-old patient who visits your physiotherapy center as he suffers from back pain. His pain started 1 year ago when he fell down the stairs during his work. At this time, his pain is localized in the lumbar and extends to the cervical region, as shown in the body diagram. The intensity of his pain is 6/10 on the numerical scale of pain (0= no pain, 10=the worst pain imaginable) 3–4 days of the week, while the rest is less. His pain appears mainly after long hours of work.

History

Mr. Raphael has no other health problems (co-morbidities) and has never been diagnosed with any other chronic condition. He has no other clinical symptoms for which further investigation is required (red flags). He does not report problems of other systems (respiratory, cardiovascular, gastrointestinal, etc.).

Appearance and Behavior

Mr. Raphael came to your physiotherapy center. While he was waiting, he remained standing. The fall from the ladder he had a year ago had not caused any fractures or any serious damage. He came to you as he feels that the situation is getting worse and the symptoms are interfering with his performance at work, which worries him greatly.

Diagnostic tests

On MRI, there is degeneration of the lumbar discs with no findings of disc projection. On the X-ray, there are no findings in the cervical and lumbar spine.

Functionality

Mr. Raphael is experiencing difficulties at his work. His pain does not allow him to work as much as before, and this worries him greatly.

Social Background

Mr. Raphael is a builder. His job includes carrying loads, going up and down stairs, and using the upper limbs. A few months ago, he undertook a building in which he made prolonged use of the compressor and believes that this aggravated his condition. His pain has forced him, he says, to be away from work several times on leave. He is currently on leave again. He lives with his wife and has one daughter. His family relies on income from his salary. In his spare time, he is engaged in gardening.
**Medication**
He takes paracetamol and sometimes Voltaren 75 mg without a prescription.
**Previous Treatments**
She has never tried physical therapy before.
**Physical Examination**
In the standing posture, there was a slight knee flexion in the right lower limb and anterior pelvic tilt. After correction, a small degree of scoliosis was observed. At the evaluation, there was a limitation of the lumbar extension range and pain. In addition, motor control deficits and increased tension in the iliac psoas were found.
**Questionnaires**
Mr. Raphael completed some questionnaires* and received the following rating:<br>Central Sensitization Inventory: 20<br>Pain Catastrophizing Scale: 35<br>Hospital Anxiety and Depression Scale Anxiety 10 and Depression: 8
**Patient Expectations**
This shows that he has few expectations other than pain relief. When asked what he would do differently if he woke up without pain tomorrow, he says he would go back to work.

S2: Vignettes Assessment Form (Experts)

Expert Code ……….

Vignette’s Code: Please enter the vignette number you intend to assess …………

Date: Please enter the assessment date ……………………….

Section A: Quantitative Assessment

Explanation

Realism: Consider if the clinical scenario presented in the vignette aligns with your real-world clinical experiences and it is believable.

Simplicity: Evaluate whether the language used is straightforward, easy to read and understood.

Clarity: Assess if the vignette is clear, not poorly worded, and not open to misinterpretation.

Completeness: Judge if the vignette provides all necessary details to make an informed clinical judgment about the type of pain.

Please rate the vignettes based on the following features (0=not at all, 4=extremely)

1. Realism: Does the vignette reflect a realistic clinical case? 0----1----2----3 ----4
   Please feel free to discuss your choice here
   ………………………………………………………………………………..
2. Simplicity: Is the vignette written in a simple way? 0----1----2----3----4
   Please feel free to discuss your choice here
   ………………………………………………………………………………..
3. Clarity: Is the vignette clear? 0----1----2----3 --- 4

   Please feel free to discuss your choice here

   ………………………………………………………………………………..
4. Completeness: Does the vignette contain sufficient information for the clinical reasoning of pain categorization? 0----1----2----3----4

   Please feel free to discuss your choice here

   ………………………………………………………………………………..

Section B: Discrimination of Vignettes

Does this vignette include a patient with nociplastic pain? Yes / No / I’m not sure

Comments (Provide detailed reasons for your answer and suggest any changes to improve the vignette for better discrimination between nociplastic and non-nociplastic pain)

…………………………………………………………………………………………………
…………………………………………………………………………………………………
…………………………………………………………………………………………………
…………………………………………………………………………………………………

S3: Vignettes Assessment Form (Raters)

Rater Code ……….

Vignette's Code: Please enter the vignette number you intend to assess …………

Date: Please enter the assessment date ……………………….

Definitions (source: https://www.iasp-pain.org/resources/terminology/)

Nociceptive Pain: Pain that arises from actual or threatened damage to non-neural tissue and is due to the activation of nociceptors. Note: This term is designed to contrast with neuropathic pain. The term is used to describe pain occurring with a normally functioning somatosensory nervous system to contrast with the abnormal function seen in neuropathic pain.

Neuropathic Pain: Pain caused by a lesion or disease of somatosensory nervous system

Nociplastic Pain: Pain that arises from altered nociception despite no clear evidence of actual or threatened tissue damage causing the activation of peripheral nociceptors or evidence for disease or lesion of the somatosensory system causing the pain.

Question 1. Has the patient's pain lasted for more than 3 months? Yes / No/ I'm not sure

Question 2. Is the distribution of the pain widespread (regional rather than discrete)? Yes / No / I'm not sure

To fulfill the criterion indicating whether the distribution of pain is widespread (regional rather than discrete), it is essential to consider the spatial involvement of the pain. Specifically, this involves assessing whether the extent of pain exceeds what would typically be expected if only nociceptive mechanisms were responsible. Additionally, the distribution of pain and hypersensitivity should extend beyond the innervation territory of any lesioned or diseased nervous structure.

These patterns of pain distribution can fulfill this criterion:

Bilateral pain/mirror pain (i.e., a symmetrical pain pattern)

Pain varying in (anatomical) location/traveling pain, including to anatomical locations unrelated to the presumed source of nociception (e.g., hemilateral pain)

Large pain areas with a nonsegmental (i.e., neuroanatomically illogical) distribution

Widespread pain (defined as pain located axial, on the left and right side of the body, and above and under the waist)

Allodynia/hyperalgesia outside the segmental area of (presumed) primary nociception

Question 3. Is there evidence that nociceptive pain is present? Yes / No / I'm not sure

Nociceptive Pain: Pain that arises from actual or threatened damage to non-neural tissue and is due to the activation of nociceptors. Note: This term is designed to contrast with neuropathic pain. The term is used to describe pain occurring with a normally functioning somatosensory nervous system to contrast with the abnormal function seen in neuropathic pain.

Question 4. Is nociceptive pain entirely responsible for the pain? Yes / No / I'm not sure

Question 5. Is there evidence that neuropathic pain is present? Yes / No / I'm not sure

Neuropathic Pain is defined as pain caused by a lesion or disease of somatosensory nervous system

Explanation:

To fulfill this criterion, it should be ensured that:

Possible neuropathic pain from the history:

History of relevant neurological lesion or disease

Pain distribution neuroanatomically plausible

Probable neuropathic pain from the physical examination:

Pain is associated with sensory signs in the same neuroanatomically plausible distribution

Definite neuropathic pain from the confirmatory test:

Diagnostic test confirming a lesion or disease of the somatosensory nervous system explaining the pain

Question 6. Is neuropathic pain entirely responsible for the pain? Yes / No / I'm not sure

Question 7. Are evoked pain hypersensitivity phenomena clinically elicitable in the region of pain? Yes / No / I'm not sure

Explanation: To meet this criterion, at least one of the following must be present.

Dynamic Mechanical Allodynia. Can be assessed by gently stimulating the skin with a brush or cotton or by light palpation and asking if the sensation produced is painful or not.

Static Mechanical Allodynia. Typically assessed by palpation using pressure of approximately 4 kilograms on the skin. Reporting pain upon this palpation is considered allodynia.

Heat or Cold Allodynia. Easily assessed by applying to the skin a metallic object maintained at room temperature (approximately 20°C). The same object can be heated with water to assess heat allodynia (approximately 40°C).

Painful after-sensations. Painful sensation that persists after assessment with any of the above tests.

Question 8. Is there a history of pain hypersensitivity in the region of pain? Yes / No / I'm not sure

Explanation: To fulfill the second criterion, a history of pain hypersensitivity to touch, pressure, movement, or heat/cold must be present. It is therefore recommended to question patients regarding their current hypersensitivity to these modalities. Patients may perceive the touch of clothing against the skin and/or the pressure from belts, handbags, and bras as unpleasant or painful. They may report hugging to be painful and/or note that it is painful to sit in a chair for any prolonged periods. Hypersensitivity to movement can be assessed by asking how habitual physical activities of moderate intensity, such as walking, affect their pain, with exercise-induced reductions of pain intensity considered to be the normal response and exercise-induced exacerbations of pain representing a sign of altered pain modulation, providing they are not better explained by a specific peripheral pathology. A history of increased pain during a cold or warm bath or shower is a typical sign of thermal hypersensitivity.

Question 9. Is there a presence of comorbidities? Yes / No / I'm not sure

Explanation: To meet this criterion, at least one of the following must be present.

The individual exhibits increased sensitivity to light and/or sound and/or smells, for example, stating "I am sensitive to bright lights" or "Certain smells, like perfumes, make me feel dizzy and nauseous."

The individual experiences sleep disturbances with frequent nocturnal awakenings, for example, stating "I feel tired and fatigued when I wake up" or "I don't sleep well" or "My legs feel uncomfortable and restless when I try to sleep at night."

The individual experiences fatigue, for example, stating "I get tired very easily during physical activity" or "I have little energy."

The individual experiences cognitive problems such as difficulty concentrating, memory disturbances, for example, stating "I find it hard to concentrate" or "I have difficulty remembering things."

Reference: Kosek, E., Clauw, D., Nijs, J., Baron, R., Gilron, I., Harris, R. E., Mico, J. A., Rice, A. S. C., & Sterling, M. (2021). Chronic nociplastic pain affecting the musculoskeletal system: clinical criteria and grading system. Pain, 162(11), 2629–2634. https://doi.org/10.1097/j.pain.0000000000002324

S4: Interpretation of Cohen's kappa for calculating agreement (adapted from McHugh, 2012)

Value of kappa	Level of Agreement
0-0.20	None
0.21-0.39	Minimal
0.40-0.59	Weak
0.60-0.79	Moderate
0.80-0.90	Strong
>0.90	Almost perfect
